# Social determinants of psychological wellness for children and adolescents in rural NSW

**DOI:** 10.1186/s12889-019-7961-0

**Published:** 2019-12-02

**Authors:** Ingrid Peters, Tonelle Handley, Karen Oakley, Sarah Lutkin, David Perkins

**Affiliations:** 1 Charles Sturt University, Bathurst, Australia; 2 Centre for Rural and Remote Health, School of Medicine and Public Health, University of Newcastle, Callaghan, NSW Australia; 3 Discipline of Child and Adolescent Health, Sydney Medical School, University of Sydney, Sydney, Australia; 4 School of Psychology, James Cook University, Townsville, Australia; 5 Centre for Rural and Remote Health, School of Medicine and Public Health, University of Newcastle, Orange, NSW Australia

**Keywords:** Rural, Children, Mental wellness, SDQ, Family

## Abstract

**Background:**

The mental wellness of children and adolescents in rural Australia is under researched and key to understanding the long-term mental health outcomes for rural communities. This analysis used data from the Australian Rural Mental Health Study (ARMHS), particularly the parent report Strengths and Difficulties Questionnaire (SDQ) measure for children under 18 years old and their reporting parent’s demographic information to compare this sample’s mental wellness scores to the Australian norms and to identify what personal, family, community and rurality factors contribute to child mental wellness as pertaining to the SDQ total and subdomain scores.

**Method:**

Five hundred thirty-nine children from 294 families from rural NSW were included. SDQ scores for each child as well as personal factors (sex and age), family factors (employment status, household income and sense of community of responding parent), community SES (IRSAD) and rurality (ASCG) were examined.

**Results:**

Children and adolescents from rural areas had poorer mental wellness when compared to a normative Australian sample. Further, personal and family factors were significant predictors of the psychological wellness of children and adolescents, while after controlling for other factors, community SES and level of rurality did not contribute significantly.

**Conclusions:**

Early intervention for children and families living in rural and remote communities is warranted particularly for low income families. There is a growing need for affordable, universal and accessible services provided in a timely way to balance the discrepancy of mental wellness scores between rural and urban communities.

## Background

Mental illness is a significant burden on the Australian health system [1]. It has been more than a decade since rates of mental illness in rural and remote communities were reported inconsistently as being similar to urban populations and up to five times higher. A report by the Australian Institute of Health and Welfare suggests that while males in outer regional areas may experience higher levels of depression and psychological distress than their urban counterparts, there are no overall inter-regional differences in mental health [2, 3]. However, more recent Australian research has shown that rural adult populations have rates of suicide up to four times higher among males [4] which may reflect a higher burden of mental illness in these regions. Given that the onset of 50% of mental illness is before the age of 14, it is imperative to consider the factors that may contribute to psychological wellness in children [5]. The rates of child psychological distress in rural Australian communities have not been adequately researched. In one study child and adolescent population mental illness rates were higher in non-metropolitan areas [6], while in 2014 suicide was the principal cause of death for children 5–17 years of age [4]. Research needs to look beyond confirming differences in mental wellness and health status by place alone and disentangle the factors contributing to this difference [7]. This indicates the need to understand what factors contribute to mental wellness within rural child and youth populations and to enable the development of appropriate early intervention strategies [8, 9].

According to the Australian Bureau of Statistics (2015b) an imbalance in service provision and difficulties in accessing services, such as a doctor, may contribute to differences in mental wellness between urban and rural populations. Child service needs in rural Australia are understudied. For adults surveyed in rural areas with an estimated high mental health service need, 47% were not connected with a service [10]. Within the few Australian studies of children, the level of remoteness alone did not account for the emotional and behavioural problems of those under 9 years old [11] or the mental health levels for adolescents 12–16 years old [12]. However a national study of 6310 families found that children and young people in non-metropolitan areas had higher rates of mental disorders [6]. Thus, the degree of rurality needs further exploration about its relationship with child and youth mental wellness.

Investigating rural mental wellness by geographic similarities alone assumes homogeneity of communities and commonalities of experience [13, 14]. Thus, a broader exploration of possible contributing factors, such as, social circumstances acting as stressors [15] for child mental wellness is required. The bioecological model postulates that there are multiple factors that impact upon a child including their community, family and individual factors [16–18]. Further, this model stipulates that variables that impact upon a parent will likely also impact upon the child. This is a useful model to explore the determinants of psychological wellness within rural children and adolescents in Australia.

Studies suggest that level or degree of remoteness is not the best variable to account for the difference between mental health levels in urban versus rural communities, rather that the key differentiating factor is the degree of community disadvantage [19]. Many international studies of the socio-economic status (SES) of communities have linked low community SES to poorer mental wellness of children [20, 21]. In the Australian context it has also been found that community SES rather than rurality predicts mental wellness in children under 9 years old [11, 22, 23]. However, there is a shortage of childhood research in Australia across the age span and so it remains unclear if this finding is true for children over 9 years old.

The SES level within the family has also been studied widely and is considered one of the most robust factors in predicting child and adolescent mental wellness both globally [20, 24] and for Australian children [6, 11, 22, 23, 25]. Evidence is emerging that the impact of family SES increases with the child’s age [25]. SES is particularly important to consider in Australian rural and remote communities as some are disproportionally affected by variable incomes dependent on cyclical factors, higher rates of unemployment and lower family household incomes than urban communities [14]. There are many ways to measure family SES including parental employment and its correlate, level of household income. The literature is robust in showing that unemployment can cause psychological distress in adults [26, 27] and there are mixed results for the role parental unemployment plays in child psychological distress [23, 28]. Exploration of the role of family SES through measures of income and parental employment needs to be undertaken to understand its impact on wellness for children in rural Australia.

Using the same sample data from this study, Kelly et al. [29] found that for adults, mental wellness, as measured by K10 scores, was moderated by personal factors including marital status, trait neuroticism and education level, as well as individual contextual factors such as sense of community. Other factors that influenced adult wellness were recent adverse events, worry about drought and exposure to rural adversity.

Other mediating factors for mental health can be seen in disadvantaged communities, such as sense of community connectedness. Globally it is accepted that an adult’s own sense of connectedness to their community is linked to their mental wellness [30–34]. Australian rural populations show a higher sense of connection to community that acts as a protective factor for adult mental wellness even when the community is experiencing adverse drought conditions [33, 35], with similar emerging evidence for adolescent populations [36, 37]. As there is a relationship between various parental factors that impact upon child mental wellness, and a high sense of community is a strong protective factor for adults in rural areas, it is prudent to consider whether parental sense of community influences child mental wellness. There is little research exploring the relationship between parental sense of community and child mental wellness.

Individual child factors such as age and sex have been shown to have a complex interaction with mental wellness throughout child development [11, 25, 38]. Due to limited longitudinal studies of child development exploring mental wellness factors, comparisons across cross-sectional studies are difficult to make due to the use of different measures of mental wellness and different age groupings in each study. Some Australian studies place age and sex as more predictive of mental wellness scores than rurality or community and family SES levels [12]. However, the mechanisms that underlie the reasons for these individual level differences are unclear.

## Hypothesis

The aim of this study is to explore the relationship between social determinants of health on the mental wellness of children in rural and remote communities in NSW. This study will; 1) compare child mental wellness in rural and regional NSW with an Australian normative sample; 2) compare rural child mental wellness across categories of remoteness; and 3) explore determinants and moderators of child mental wellness. It is hypothesised that the relationship between child mental wellness and rurality will be moderated by personal factors of age and gender, and familial factors including family SES measured by parental employment and income, and parental sense of community and community SES factors.

## Method

### Participants

The Australian Rural Mental Health Study (ARMHS) is an Australian longitudinal population study examining the determinants of mental wellness in rural and remote communities. Data was collected in 2006–2009 from adults living in New South Wales (NSW) excluding major metropolitan zones (e.g. Sydney and Newcastle) [39]. All invited participants were over 18 years old and randomly selected from the Australian Electoral Roll (a list of all adult Australian residents who are registered to vote). A random number generator was used to identify potential participants. Those who were selected by this random number generator received a phone call to inform them about the study, and ask their interest in participating. A household sampling frame was used, whereby people who were selected for participation were also mailed a survey for each other adult residing in their house; they were also mailed a survey for each child in the house, to be completed by the parent or guardian. People in special dwellings such as hospitals and prisons, those without an identifiable telephone number, non-English speaking members of a household, and those with hearing impairments that made obtaining phone consent difficult were excluded. Participants over 65 years were briefly screened for cognitive status using the Telephone Interview for Cognitive Status (TICS-M) and those with a score of < 17 were excluded.

**Fig. 1:**
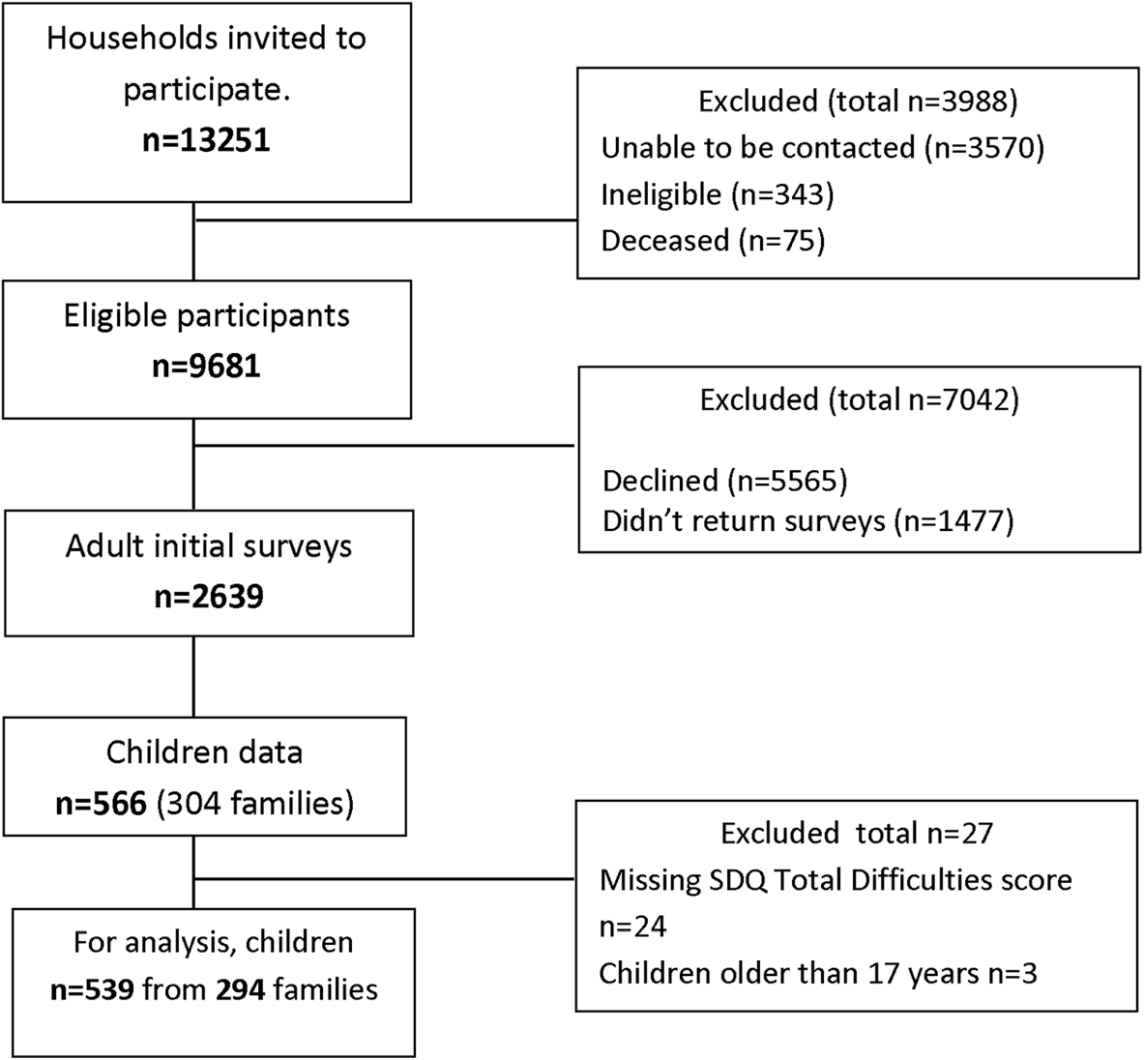
Participant selection and exclusion for current study

Information about the study and surveys were mailed, with up to five follow up telephone contacts. The data was collected at baseline with follow up at 3 years for children and the parent who completed the child measures. Participant numbers in the current analysis are provided in Fig. 1. The project was approved by the Human Research Ethics Committees of participating institutions.

### Instruments

#### Current child psychological wellness

The Strengths and Difficulties Questionnaire -Parent Report (SDQ, [40]) is a 25-item questionnaire for parents or carers to report on how they perceive their child’s level of functioning as a mechanism to assess psychological wellness and distress in children. There are versions for children aged 4–10 and 11–17 years old. The SDQ has five subscales: emotional symptoms, conduct problems, hyperactivity-inattention, peer problems and prosocial behaviour. Each item is scored 0, 1 or 2, with somewhat true always scoring 1 and not true and certainly true scoring 0 or 2 depending if the item is a strength or difficulty. The absence of prosocial behaviour is indicated by lower scores whereas higher scores on the other domains indicate increased problems. The SDQ also calculates a Total Problem score which includes four of the domains above, excluding prosocial behaviour. The maximum Total Problem score is 40, with a higher score indicating increased problems. The SDQ is psychometrically sound with good internal consistency (Cronbach α: 0.73) and re-test reliability (0.62) [41] and is widely used as a screening tool for psychological wellness in children [42]. Further, SDQ scores in the 90th percentile have predictive validity for independent psychiatric disorders for the SDQ parent, teacher and youth scales [41].

#### Family SES

Family SES was measured by two independent characteristics: parental employment status, and household income. Parental employment status was self-reported by the parent responding to the survey. Household income was also self-reported by the responding parent, as the combined income of all members of the household, from all sources (including benefits, pensions, and superannuation) [39].

#### Parental sense of community

The Sense of Community Index, a 12-item true or false self-report measure that depicts a person’s sense of connection to a place or community [43] was administered to the responding parent. This is a psychometrically adequate tool with higher score indicating stronger attachment and connection to the community [44].

### Rurality factors

#### Community SES

The Index of Relative Socio-Economic Advantage and Disadvantage (IRSAD, [45]) was used to represent the level of advantage and disadvantage for an area through the families’ reported postcode. The IRSAD encapsulates variables such as family income, mortgage levels, education levels, household overcrowding and vehicle access, and then assigns a decile based on these factors. A higher IRSAD score indicates relatively less disadvantage and more general advantage. A lower score indicates relatively more disadvantage and less general advantage. The IRSAD formula used for this study was based on Australian Bureau of Statistics 2006 Census of Population and Housing data to match when the participant data was collected.

#### Level of Rurality

The Australian Standard Geographic Classification (ASGC) categorises the level of remoteness based on the Accessibility/Remoteness Index of Australia Plus (ARIA+), a measure of the level of remoteness in road distance from required services within Australia [46]. The ASGC groups the ARIA+ into five categories: major cities, inner regional, outer regional, remote, and very remote. This study uses the last four categories as major cities were not included in the sample.

#### Data analysis

Data entry, cleaning and analysis was performed using Statistical Package for Social Sciences version 25 statistical software [47].

Analysis primarily reported SDQ as a continuous variable as this gave clearer indications of changes. When SDQ scores are grouped it was into clinical significance bands of normal, borderline and abnormal, based on the Australian norms by Mellor [48].

Due to non-normality of the data, the SDQ subscale and total scores, and parental Sense of Community scores were transformed to standardised scores for further analysis.

Household income was grouped into three categories based on exploratory analysis which grouped the participants into equal income thirds and based on appropriate statistical comparison of income groups on SDQ means. This matched the Australian Taxation Office personal income tax brackets for 2010–2011 being split into thirds. Low income includes nil income to $37,000, medium income $37,001 to $80,000 and high income above $80,000.

Employment status of the parent completing the child measures was spilt into three groups based on appropriate statistical comparison of SDQ means across the original five employment categories. There were no significant differences in SDQ scores across those who were not working (unemployed (*n* = 19), not working due to illness/disability (*n* = 13) and retired (*n* = 1); this was therefore collapsed into one group. The remaining groups were employed and unemployed.

Demographic differences were examined using ASGC Chi square, Fisher’s Exact test, independent sample t-tests, ANOVAs and Kruskall-Wallis statistics as appropriate.

Comparisons were conducted of the current rural SDQ sample with Australian norms [48] using independent sample t-tests.

The relationship between child psychological wellness (using the SDQ subscales and total score) and personal factors (sex and age), family factors (employment status and sense of community of responding parent and household income), community SES (IRSAD) and rurality (ASCG) were examined by conducting Pearson’s correlations, t-tests, and Chi square analyses as appropriate. For employment status where no correlation could be conducted, an ANOVA was used.

The influence that each level of factors impacted upon SDQ scores was analysed using Multiple Linear Regressions and hierarchical analysis with variables were entered in four steps: step 1, personal factors (child age, child gender); step 2, Parental/Family factors (parental employment status, household income, and parental sense of Community); step 3, community SES (IRSAD); and step 4, level of rurality (ASGC). This model was used for each SDQ subscale and total score. The order of factors entered was based on postulated order of influence from the relevant literature on child development, following the method of Kelly et al. [29], although separating community SES and level of rurality into separate steps based on the existing literature and hypotheses.

A moderator analysis was conducted to examine whether ASGC interacted with age or parental sense of community in influencing children’s psychological wellness measured through SDQ subscales and total scores.

For all analyses, statistical significance was set at *p* ≤ 0.05.

## Results

The SDQ parent report forms were completed by 294 separate families for 539 children. Within the sample 169 parents completed SDQ forms for more than one child in their family (Table 1). Most parents who completed the SDQ forms were female (86.4%). Children were between 4 and 17 years old (*M* = 10.96 and *SD* = 3.8) with a roughly equal split in sex (female *n* = 197, 49.9%).

**Table 1:** Family Demographics for analysis sample

Children per family	Families	% of total
1	125	42.5
2	113	38.4
3	39	13.3
4	15	5.1
5	1	0.34
6	1	0.34

Table 2 presents the demographic information for the sample population as separated into remoteness categories. It should be noted that there was some missing data on some variables for some participants. Analysis for expected people per remoteness category for variables are included.

**Table 2:** Participant Demographic Characteristics by ASGC (remoteness) category

Variable	Australian Standard Geographic Classification (ASGC)				*p* value	Total (*n* = 539)^a^
	Inner Regional (*n* = 164)^a^	Outer regional (*n* = 205)^a^	Remote (*n* = 118)^a^	Very remote (*n* = 52)^a^		
Child age Group						
4–10 years	64 (25.6%)*, (*n* = 43, *m* = 7.33, *SD* = 2.01)	90 (36%), (*n* = 59, *m* = 7.15, *SD* = 2.024)	71 (28.4%)*, (*n* = 52, *m* = 7.19, *SD* = 2.11)	25 (10%), (*n* = 15, *m* = 6.93, *SD* = 1.71)		250 (46.8%), (*n* = 169, *m* = 7.19, *SD* = 2.02)
11–17 years	97 (34.2%)*, (*n* = 75, *m* = 13.72, *SD* = 1.91)	114 (40.1%), (*n* = 89, *m* = 14.09, *SD* = 1.84)	47 (16.5%)*, (*n* = 42, *m* = 13.14, *SD* = 2.04)	26 (9.2%), (*n* = 21, *m* = 13.90, *SD* = 1.92)		284 (53.2%), (*n* = 227, *m* = 13.78, *SD* = 1.91)
Total	161 (30.1%)	204 (38.2%)	118 (22.1%)	51 (9.6%)	.006	534 (99.07%)
Child gender						
Female	56 (28.1%)	83 (41.7%)	46 (23.1%)	14 (7%)	.206	199 (36.9%)
Male	65 (32.0%)	67 (33.0%)	48 (23.6%)	23 (11.3%)		203 (37.7%)
Parental Employment Status						
Employed	115 (27.9%)*	160 (38.8%)	90 (21.8%)	47 (11.4%)*		412 (76.4%)
Unemployed	10 (30.3%)	18 (54.6%)	4 (12.1%)	1 (3.0%)		33 (6.1%)
Home/Study	33 (41.3%)*	20 (25.0%)*	24 (30.0%)	3 (3.8%)*		80 (14.8%)
Total					.003	389 (72.2%)
Household Income						
Low	35 (27.6%)	42 (33.1%)	42 (33.1%)*	8 (6.3%)		127
Medium	48 (34.8%)	60 (43.5%)	19 (13.8%)*	11 (8.0%)		138
High	33 (26.6%)	45 (36.3%)	29 (23.4%)	17 (12.7%)*		124
Total					.005	389 (72.2%)
Community SES categories						
Low	34 (14.7%)*	102 (44.2%)*	78 (33.8%)*	17 (7.4%)	<.001	231 (44%)
Medium	98 (40.2%)*	77 (31.5%)*	34 (13.9%)*	35 (14.3%)*		244 (46%)
High	28 (51.9%)	20 (37.0%)	6 (11.1%)*	0*		54 (10%)

*significantly different number of participants in group than expected

^a^This was the total n for each group, however as full data was not available for each variable, the result is based on the available data

### SDQ sample scores compared to Australian normative data

SDQ for the current sample’s internal consistency was moderate (Cronbach’s alpha 0.68). Table 3 shows comparisons between the current non-urban sample and the Australian norms made across remoteness categories and SDQ domains. The total problem scores for the current sample had a mean difference of 1.05 which was significantly higher than the normative sample (Welch’s t(1221.61) = 3.37, *p* = .001). There were significantly greater conduct problem scores in the rural sample with a mean difference of 0.55 when compared with the normative data (t(1447) = 6.44, *p* < .001). Similarly, the peer problems mean was 0.51 higher in the current sample than the Australian normative sample (Welch’s t(1230.08) = 5.28, *p* < .001). Conversely, the current sample also had significantly better prosocial skills than the Australian normative data with a mean difference of − 0.26 (Welch’s t(1034.12) = − 2.66, *p* = .008) [48]. Greater conduct problem scores than the normative data were also noted across all the remoteness regions (see Table 3). Children in inner regional areas and remote regions also had greater total difficulties and peer problems (see Table 3). Those in very remote regions also had greater emotional symptoms and peer problems (see Table 3). Those in inner regional areas had greater social skills than the normative data.

**Table 3:** SDQ Results by ASGC category

	Australian Standard Classification (ASGC) category				Whole Sample
	Inner Regional (*n* = 164)	Outer regional (*n* = 205)	Remote (*n* = 118)	Very remote (*n* = 52)	Mean (*SD*) total sample
Total Difficulties Mean (*SD*)	9.60 (6.14)*	8.47 (5.03)	10.24 (5.77) **	8.98 (4.07)	9.25 (5.50)**
Emotional Symptoms Mean (*SD*)	2.08 (2.18)	1.89 (1.83)	2.05 (2.11)	1.61 (1.62)*	1.96 (1.99)
Conduct Problems Mean (*SD*)	2.02 (1.47)**	1.84 (1.50)*	4.47 (1.61)**	2.00 (1.36)*	2.05 (1.52)**
Hyperactivity- inattention Mean (*SD*)	3.38 (2.75)	2.89 (2.29)	3.29 (2.44)	3.06 (2.03)	3.14 (2.45)
Peer Problems Mean (*SD*)	2.13 (1.79)**	1.85 (1.68)	2.41 (1.55)**	2.33 (1.76)*	2.10 (1.70)**
Prosocial Behaviour Mean (*SD*)^a^	7.93 (1.97)*	8.04 (1.78)	8.22 (1.83)	7.94 (1.71)	8.04 (1.84)*

Note: significant differences between the current sample and Mellor sample means found at *p < 0.05, **p < 0.001

^a^lower score indicates higher problem levels

### SDQ scores and remoteness groups

There was a significant difference between the number of people within an SDQ domain or category across all remoteness areas in the Total Difficulties Score (Welch’s F (3, 202.76) = 2.95, *p* = 0.034), Peer Problems Score (Welch’s F (3, 535) = 3.03, *p* = 0.028) and SDQ Conduct Problem scores (F (3, 535) = 4.53, *p* = 0.004). Significant differences (see Table 3) were found between outer regional and remote communities, with those in outer regional areas scoring significantly lower than those in remote areas for each of these outcomes. The magnitude of these differences varied, with outer regional residents scoring an average of 2.63 points lower than remote residents for Conduct Problems; for Peer Problems there was only a 0.56 point difference between the groups.

### Variables that contribute to SDQ scores differences

Analyses of the relationship between personal, family, community SES and rural factors are presented in Table 4.

**Table 4:** Predictors of psychological wellness based on four-step hierarchical regression analysis (*n* = 284)

	SDQ Total Problem Score		SDQ Emotional Symptoms		SDQ Conduct Problems		SDQ Hyperactivity-Inattention		SDQ Peer Problems		SDQ Prosocial Behaviour	
	*r*^2^/β^a^	*r*^b^	*r*^2^/β^a^	*r*^b^	*r*^2^/β^a^	*r*^b^	*r*^2^/β^a^	*r*^b^	*r*^2^/β^a^	*r*^b^	*r*^2^/β^a^	*r*^b^
1. Personal Factors	0.04***		0.01		0.11***		0.05***		0.03**		0.03**	
Age	− 0.16**	− 0.15	0.12*	0.12	−0.32***	− 0.31	− 0.14*	− 0.19	− 0.17**	− 0.16	− 0.04	−0.04
Sex (1 = male)	− 0.15**	−0.15	− 0.05	−0.05	− 0.10	−0.10	− 0.20**	−0.19	− 0.07	− 0.07	0.20**	0.19
2. Family Factors	0.14***		0.07***		0.16**		0.09**		0.01***		0.03	
Household Income												
Low vs high	0.17**	0.14	0.17*	0.14	−0.15*	0.13	0.04	0.04	0.17*	0.14	−0.03	−0.02
Medium vs high	0.14*	0.12	0.11	0.09	0.17*	0.14	0.15*	0.12	−0.02	− 0.02	0.05	0.04
Sense of Community	−0.26***	−0.25	− 0.21***	−0.19	− 0.17**	−0.16	− 0.19**	−0.18	− 0.19**	−0.18	0.09	0.09
Employment status												
Unemployed vs employed	0.08	0.08	0.04	0.03	0.06	0.06	0.06	0.06	0.09	0.09	−0.05	− 0.05
Unemployed vs home	− 0.53**	− 0.14	− 0.14*	− 0.13	− 015**	− 0.14	−0.10	− 0.09	−0.06	− 0.06	0.05	0.05
3. Community SES	0.14		0.07		0.16		0.09		0.09		0.03	
IRSAD	−.000	−0.00	−0.06	− 0.05	0.06	0.06	0.00	0.00	0.00	0.00	−0.01	− 0.01
4. Rurality	0.13		0.06		0.16		0.09		0.09		0.02	
ASGC groups												
Outer Regional vs Inner Regional	−0.06	−0.05	−0.01	−0.01	− 0.02	−0.02	− 0.09	−0.07	− 0.05	−0.04	0.03	0.03
Remote vs Inner Regional	−0.01	0.01	−0.01	−0.01	0.09	0.07	−0.02	−0.02	− 0.01	−0.01	0.08	0.06
Very Remote vs Inner Regional	−0.02	−0.02	− 0.07	−0.06	0.01	0.01	−0.04	−0.04	0.04	0.04	0.02	0.02

Note. ^a^ model values are adjusted *r*^2^, individual variables are β;^b^part correlations; *p < 0.05, **p < 0.01, *** *p* < 0.001

Personal factors contributed significantly to the total SDQ score and all SDQ subscales, except emotional symptoms, indicating that there was generally poorer mental wellness for males and younger participants.

Family factors contributed significantly to total SDQ scores and all SDQ subscales except prosocial behaviour. Mental health was poorer for those in families with lower household incomes, lower parental sense of community scores, and with unemployed parents.

After controlling for personal and family factors, community SES did not significantly contribute to the variance for the SDQ scores. Furthermore, after controlling for personal, family, and community factors, the relationship between remoteness and SDQ scores was no longer significant.

Moderator analyses examining impact of the interaction between remoteness and sense of community on the SDQ measures revealed no effect. Similarly, there was no significant impact of the interaction between remoteness and age on SDQ scores.

## Discussion

There is little evidence about the social determinants of psychological wellness for young people within rural and remote communities. This study aimed to identify the social determinants of health that impact on the psychological wellness of children in rural regions of NSW, Australia. This study established that children and adolescents in rural and remote communities of NSW have poorer psychological wellness than the general Australian population. Consistent with expectations we found that personal and family factors were significant predictors of the psychological wellness of children and adolescents, while after controlling for other factors, community SES and level of rurality were not significant contributors.

This is the first study to show that children and adolescents in rural and remote NSW have poorer psychological wellness when compared to normative data from Mellor’s (2005) general Australian population study. It also provides evidence of generally lower levels of psychological wellness in children and adolescents in regions that are rural, with remote regions having the worst overall scores. This concurs with research in adults showing poorer mental health in rural and remote communities of Australia [2].

The initial analysis confirmed broad differences in this rural population’s psychological wellness, as well as poorer results for children within remote communities. Rurality was not significant in the hierarchical regression. Thus, whilst differences in psychological wellness are clear by rurality category, either it is not the rurality itself that impacts on psychological wellness or the ASGC is not a sufficiently sensitive measure. This finding is similar to prior research with adults in rural communities [29] and suggests the need for investigation of a wider scope of childhood psychological well-being influences at multiple levels of the Bronfrenbrenner’s bioecological model.

Community SES did not impact on psychological wellness within this sample. This finding should be considered cautiously due to the uneven distribution of participants in the IRSAD categories and differs from national and international research which suggests community SES is a significant factor contributing to child and adolescent mental wellness [20, 21, 24]. Household income was seen to be connected with some SDQ results and warrants further investigation.

In this study child psychological wellness was not primarily related to rural contextual factors of community relative advantage, disadvantage and degree of rurality. Personal factors of age and gender, and familial factors including family SES as measured through parental employment and income, and parental sense of community had the greatest significant contribution to child and adolescent wellness.

The personal factors of age and gender have a significant and complex influence in child and adolescent psychological wellness in both rural and remote communities and more broadly. This study highlighted that similar to the general population [12, 49] personal factors of younger age and male gender are significant factors in childhood wellness in rural regions of NSW. Further, age and gender are more influential than factors of rurality and community SES. While this does not give insight into the presence of a greater degree of problems in rural and remote locations, it does indicate that the knowledge regarding the personal factors for child mental wellness apply to rural and remote locations, which should influence surveillance, prevention, early intervention and treatment policies and practices in these regions.

The impact of family factors such as household income level and factors of SES influencing child psychological wellness in Australia are consistent with the emerging Australian evidence for rural and remote children under 9 years old [11] and the general Australian child population of four to 7 years old [22, 23].

Additionally, this study included parental sense of community in family factors and found this to be a strong contributing factor, with higher parental sense of community associated with higher levels of child mental wellness. This was evident across SDQ total scores as well as all subdomains except prosocial behaviour. There is strong national and international evidence for the relationship between psychological wellness and connectedness to one’s community for adult populations [30–33] and evidence is emerging that this is consistent for rural adolescent populations where self-reported sense of community acts as a moderator of mental wellness [35, 37]. There is very little research on the role that parental sense of community plays directly in child psychological wellness and how to enhance parental sense of community to benefit children [50]. In retrospective adult studies there is evidence that strong sense of community can act as a protective factor [51, 52]. There is also evidence of connections with supportive and “healthy” adults and parents to increase resilience amongst children [53]. Future studies could develop a child’s sense of community measure. Consideration should be given to understanding the connection between parental sense of community and the impact this has on children. Through understanding the impact of sense of community on both the child and parent individually and in connection with each other interventions can be designed to enhance community connectedness as a protective factor for families.

The three components explored in this study; personal, family and wider community; reflect the bioecological theory of Bronfenbrenner [16, 17] about understanding a person within their context. This study found the critical importance of personal and family factors, but not community factors i.e. degree of rurality or relative disadvantage. It is plausible that other community factors that were not examined including environmental stressors, such as drought, flood or fires, may also influence child and adolescent wellness and these need further exploration.

The study findings of lower psychological wellness in rural and remote children and adolescents, and that personal and family factors are more influential than rural contextual factors are consistent with the findings for adults in the Australian Rural Mental Health Study [29].

These consistent findings of personal and family factors influencing psychological wellness of children and adults in rural and remote locations also match what is known about the influences on psychological wellness in the general population [19, 54]. However, this research raises an interesting question about reasons for the disparity between urban and rural communities’ mental wellness and warrants further investigation of broader factors suggested by the bioecological model. One study that compared the research from Australia, New Zealand, Canada, the UK and the USA found that levels of rurality did not have a direct correlation to morbidity [55]. Rather, it was found that rurality levels can exacerbate the impact of other risk factors which combine to create higher levels of disadvantage. This study recommended looking at the broader context of rural living. While it did not focus on mental wellness, its findings on health may warrant further consideration. Another factor to consider is the poorer engagement with services in rural Australian communities, with one study finding that 47% of adults estimated to have high service needs were not connected to a service [10]. This could also be true for children and adolescents, more so if they require a specialist service. Further, the imbalance of service provision between major cities and very remote areas is likely to impact adversely on child wellness [56].

### Limitations

This study is the first to explore child and adolescent wellness throughout rural and remote regions of one Australian state and included only children who reside at home rather than those boarding away for educational purposes. Therefore, whether these findings extend to wider rural and remote population needs to be established. While the results are limited to this population, the importance of these findings cannot be underestimated. Since no comparison urban sample was collected the findings in this study were compared to a published Australian normative sample. While methodological differences between samples may account for some of the differences in wellness scores, it is notable that much research in psychology similarly compares their findings to those of published norms. Since the data was collected within a larger cohort study conducted 10 years ago, all findings have been compared with contemporary not current values.

Other limitations include a low response rate, albeit one that is congruent with other rural based population-based surveys [57]. The current study used parent observational reports for child wellness and more nuanced future research may benefit by including child and teacher report measures for both wellness (SDQ) and social support [37, 58, 59]. Further inclusion of personal demographic information such as Aboriginal and Torres Strait Islander or Culturally and Linguistically Diverse identifiers may help to further assess differences in mental wellness levels [12, 60].

### Future research

As there is such little Australian specific evidence about children from regional and remote communities, further research is needed to deepen the understanding of factors influencing their mental wellness. Boosting the psychological wellness of rural children and teens may help to address the differences in mental wellness between rural and urban adults.

Many other family factors should be included in future studies including parental mental illness and its impact on child psychological wellness within a rural context [61], and other broader family stressors [62].

As SDQ is a broad measure of wellness [41], to understand about more specific aspects of childhood psychological wellness more extensive questionnaires such as the Child Behaviour Checklist and Behaviour Assessment System for Children would be useful to map wellness concerns to DSM criteria and to more specifically understand the components of lower psychological wellness scores in rural communities.

## Conclusion

This is the first time that poorer psychological wellness in children and adolescents from rural and remote communities has been demonstrated in Australia and has implications for understanding psychological wellness and intervention needs for children in these communities. In NSW rural communities a child’s psychological wellness is statistically significantly lower than the Mellor’s (2005) Australian norms on the parent report SDQ measure. This study found that rural contextual factors did not significantly contribute to SDQ scores. Rather, personal and family factors had a greater and significant contribution to wellbeing.

This important finding requires further investigation. It would be prudent to consider the implications of this finding to support early intervention for children and families in rural and remote communities in the hope of preventing worsening mental health outcomes with time. Government policies and programs may need to address the strengthening of rural communities to benefit child wellness. Further, policies to minimise the impact of low-income families’ household stress should be considered by policy makers through initiatives to support families and children with poor psychological well-being, with affordable, universal, accessible and timely services.

## Data Availability

The datasets used and/or analysed during the current study are available from the corresponding author on reasonable request.

## Abbreviations

ARIA+: Accessibility/Remoteness Index of Australia Plus
ARMHS: Australian Rural Mental Health Study
ASGC: Australian Standard Geographic Classification
IRSAD: Index of Relative Socio-Economic Advantage and Disadvantage
NSW: New South Wales
SDQ: Strength and Difficulties Questionnaire
SES: Socio Economic Status
